# When Aortic Stenosis Is Not Alone: Epidemiology, Pathophysiology, Diagnosis and Management in Mixed and Combined Valvular Disease

**DOI:** 10.3389/fcvm.2021.744497

**Published:** 2021-10-15

**Authors:** Francesca Mantovani, Diego Fanti, Elvin Tafciu, Simone Fezzi, Martina Setti, Andrea Rossi, Flavio Ribichini, Giovanni Benfari

**Affiliations:** ^1^Cardiology, Azienda USL- IRCCS di Reggio Emilia, Reggio Emilia, Italy; ^2^University of Verona, Section of Cardiology, Verona, Italy

**Keywords:** aortic stenosis, aortic regurgitation, mitral stenosis, mitral regurgitation, tricuspid regurgitation, mixed valve disease, combined valve disease, echocardiography

## Abstract

Aortic stenosis (AS) may present frequently combined with other valvular diseases or mixed with aortic regurgitation, with peculiar physio-pathological and clinical implications. The hemodynamic interactions between AS in mixed or combined valve disease depend on the specific combination of valve lesions and may result in diagnostic pitfalls at echocardiography; other imaging modalities may be helpful. Indeed, diagnosis is challenging because several echocardiographic methods commonly used to assess stenosis or regurgitation have been validated only in patients with the single-valve disease. Moreover, in the developed world, patients with multiple valve diseases tend to be older and more fragile over time; also, when more than one valvular lesion needs to address the surgical risk rises together with the long-term risk of morbidity and mortality associated with multiple valve prostheses, and the likelihood and risk of reoperation. Therefore, when AS presents mixed or combined valve disease, the heart valve team must integrate various parameters into the diagnosis and management strategy, including suitability for single or multiple transcatheter valve procedures. This review aims to summarize the most critical pathophysiological mechanisms underlying AS when associated with mitral regurgitation, mitral stenosis, aortic regurgitation, and tricuspid regurgitation. We will focus on echocardiography, clinical implications, and the most important treatment strategies.

## Background

Multivalvular disease (MVD), defined as the combination of stenotic or regurgitant lesions of two or more cardiac valves, is increasingly frequent in clinical practice, presenting in 10% of patients undergoing valvular surgery. Nevertheless, it is still poorly studied (1). Aortic stenosis (AS) is the second most common valvular disease in the western world after mitral regurgitation (MR), affecting 2% of the population between 65 and 75 years and 6% of those older than 75 years (2), and is frequently associated combined with other valvular disease or mixed with some degree of aortic regurgitation (AR), with physio-pathological and clinical implications.

Nowadays, degenerative etiology comprises the vast majority of cases; however, when associated with other valve diseases, rheumatic heart disease should be considered as it is the most common cause of MVD worldwide; typically, rheumatic heart disease affects younger patients (2) and has a faster progression than the degenerative counterpart, involving almost invariably the mitral valve (3). The clinical impact of combined valve disease depends on hemodynamic interactions between the valve lesions and, more specifically, on the severity, combination, and chronicity of each valvular defect. All these factors may alter loading conditions and ventricular function with relevant consequences when assessing the severity of valvulopathies, currently based on the concept of excess mortality threshold. Indeed, in some settings, apparently non-severe lesions may lead to severe hemodynamic imbalance when combined with other valvular defects with important clinical implications (4). Methods commonly used for the quantification of stenosis or regurgitation have been validated in patients with single-valve disease, and until today the major treatment trials often excluded concomitant relevant valvular disease. In addition, one of the most pivotal issues in the management of patients with MVD is to identify the optimal timing for intervention when the benefits of the procedure most outweigh the risks, considering that these patients generally have many comorbidities and that surgery in these patients is associated with high operative mortality (5). Expertise in cardiac surgery, transcatheter interventions, and cardiac imaging is critical in this field. In the past, the surgical indication represented the crucial decision moment in which cardiologists used to indicate whether one or more valves needed to be treated. Now things have changed, and thanks to percutaneous procedures, it is possible to treat one valve at a time, evaluating the new hemodynamic balance from time to time and giving the opportunity better to understand the physiopathology of combined valvulopathies (6).

The aim of this review is to summarize the most important pathophysiological mechanisms underlying AS when associated with MR, MS, AR, and tricuspid regurgitation (TR). We will focus on echocardiography, clinical implications, and the most important treatment strategies. Although helpful in diagnosis and prognosis in selected cases of multivalvular diseases, this review does not discuss the application of multimodality imaging (TC, MRI, stress echocardiography).

## Aortic Stenosis and Mitral Regurgitation (AS-MR)

According to different studies, MR is reported in 20–80% of patients with AS. In the PARTNER trial, about 20% of patients undergoing transcatheter or surgical aortic valve replacement for severe AS presented concomitant moderate to severe MR (7). In most cases, MR in these patients is not evaluated severely, and until the last decades, the pathophysiological and clinical interaction between these two entities was not fully understood. Things have changed as some studies described essential consequences in morbidity and mortality (8).

Functional MR is present in 63% of patients with AS (9) and is likely to improve after aortic valve replacement more significantly than degenerative MR. Thus, a careful evaluation of the MR mechanism is crucial for the decision of whether a simultaneous operation on the mitral valve is necessary (10) considering that mixed mechanisms are frequent, especially in older patients with heavy mitral calcification and wall motion defects, not uncommon in patients with AS. By note, attention should be paid to obstructive hypertrophic cardiomyopathy that may present with high gradients in the LVOT and MR secondary to systolic anterior movement (11).

AS is a condition classically associated with increased afterload, but when concomitant MR is present, the left ventricle is somewhat larger as volume overload is also present. MR reduces afterload and reduces stroke volume significantly, causing a low flow-low gradient condition with the risk of underestimating the severity of AS (12, 13). Rossi et al. showed that MR was generally mild in severity when functional in origin, with an effective regurgitant orifice (ERO) smaller than 0.2 cm^2^ in 91% of the cases. Anyway, the presence of MR may falsely underestimate the transvalvular aortic valvular gradient even if volumetrically not relevant (9).

An ERO as low as 0.2 mm^2^ carries a probability of 30% developing a low-flow low-gradient condition, with the risk increasing with the MR severity (12). Simultaneously, the presence of MR reduces total afterload, increasing the ejection fraction which may hide subclinical myocardial dysfunction (14).

The impact of functional MR in AS was also studied with an artificial model that allowed to regulate the flow and the aortic valve area, demonstrating that both the mean pressure gradient and maximal velocity are significantly reduced by a reduction of forward stroke volume from concomitant severe MR. However, the functional aortic valve area appeared to be a reliable even in case of severe MR (15).

Subsequently, these patients may also develop atrial fibrillation with preload impairment due to loss of the “atrial kick,” a poorly hemodynamically tolerated condition in these patients *per se* associated with left atrial enlargement and MR progression. Generally, symptoms do not always correlate with the AS severity and LV function (16, 17); concomitant MR is associated with poorer outcomes (9).

**Figure 1:**
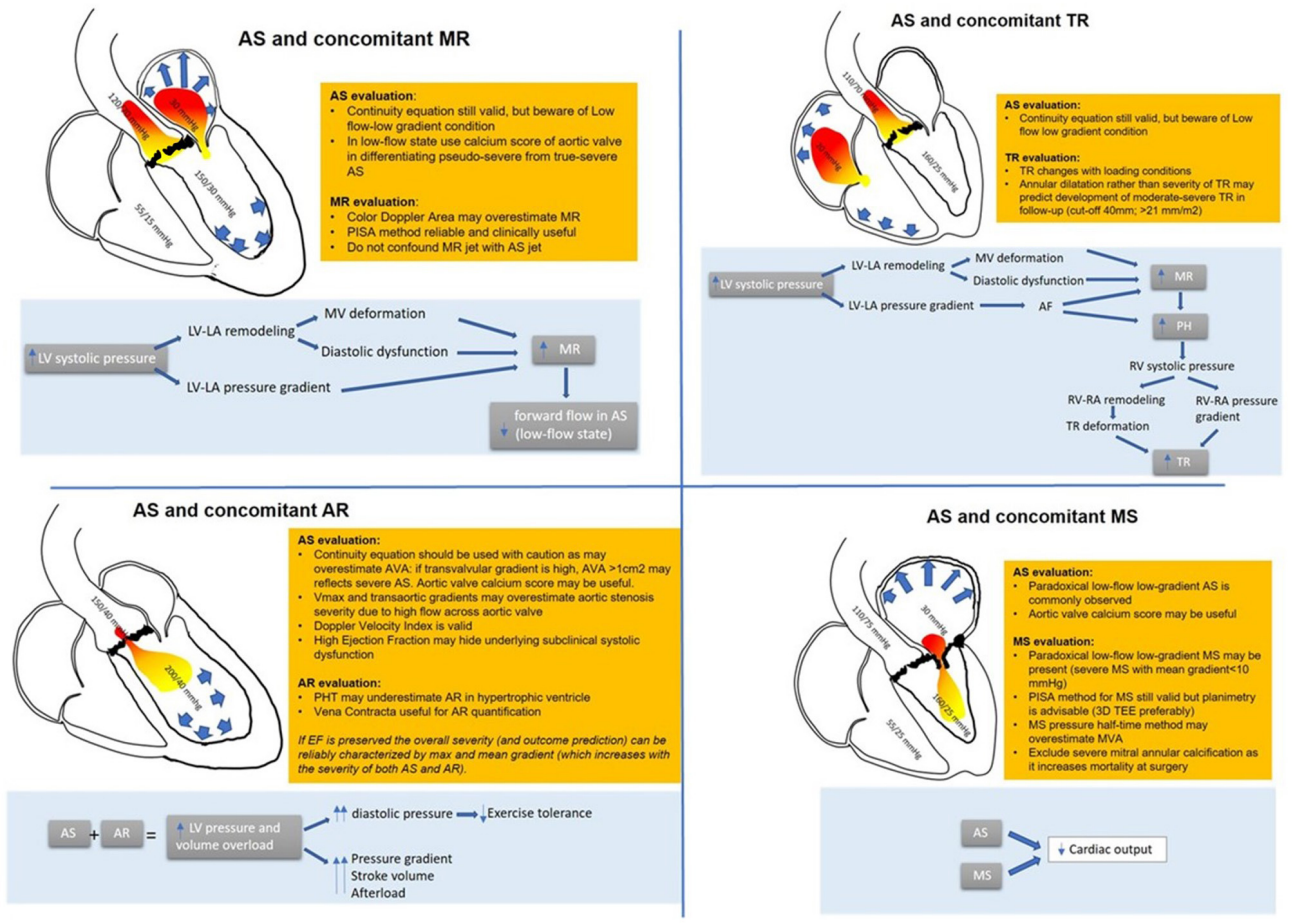
Combination of AS and other valvular heart disease: hemodynamic interactions and diagnostic pitfalls and tricks.

Generaux et al. propose a new staging system of AS that attributes severity considering the presence of concomitant MR, impaired left ventricular function, pulmonary hypertension, and TR (18). This system confirmed that MR provides incremental predictive value in patients with asymptomatic moderate to severe AS undergoing surgical and percutaneous aortic valve replacement (AVR) (19). An incremental prognostic value over clinical characteristics was shown by incorporating left ventricular global longitudinal strain into the staging classification (20). Pighi et al. recently reported that cardiac damage classification is significantly associated with a higher incidence of acute kidney injury following percutaneous AVR and that it is an independent predictor of 12-month all-cause mortality only in patients with advanced stages of extravalvular cardiac damage (21) (Figure 1).

### Diagnostic Implications

When assessing AS and MR, a quantitative approach is useful: vena contracta is reliable to assess MR as it does not depend on afterload (22). In patients with MR associated with AS, the ERO calculation with the proximal isovelocity area method proved reliable. ERO correlates with mortality and predicts heart failure (22), but regurgitant volume calculation may be falsely overestimated for a given ERO due to the higher intraventricular pressure. The color flow area may also not be proportionate to the severity of MR due to the high transmitral regurgitation jet velocity (23).

Concerning AS severity evaluation, the low flow condition produces falsely low transvalvular gradients. Functional aortic valve area calculation is helpful in patients with AS and MR with good correlation with outcomes (24).

A small retrospective study compared echocardiography and invasive catheterization parameters; for a given aortic valve area calculated with the continuity equation, the presence of significant MR does not reduce the peak transvalvular velocity as much as the calculated mean gradient. This may be explained by the squared relationship between velocity and gradient, with a small difference in velocity having a significant impact on calculated pressure (25).

The combination of AS and MR put some technical and diagnostic challenges. It must be accounted that in some cases, the high-velocity MR jet may be mistaken for the AS jet, especially in the apical view: however, MR jet is longer in duration, starting with mitral valve closure and continuing until mitral valve opening, and has a different shape in Doppler CW especially in the case of chronic MR (10).

Finally, a 3D echo evaluation of the mitral valve and aortic valve calcium score by multidetector computed tomography may be helpful in those cases where dobutamine stress echocardiography is inconclusive or contractile reserve is absent (26) (Figure 1).

### Management

The presence of moderate to severe MR in patients undergoing transcathether aortic valve implantation (TAVI) is associated with higher mortality and poorer outcomes in rehospitalization for heart failure (8, 27).

After TAVI, MR improves by at least one grade in almost 80% of patients with severe MR and 66% of patients with moderate MR (28). Patients with degenerative MR have poorer outcomes than patients with functional MR (29). Improvement of the MR at 30 days is reported in 69% of patients undergoing surgical AVR and 58% after TAVI (30). However, in some cases, MR may even worsen after the operation. Some conditions seem to be associated with lack of improvement of MR following TAVI; some of these are low baseline aortic gradient, pulmonary hypertension, degenerative MR, deep positioning of the implanted valve, heavy annulus, atrial fibrillation, and mitral annular dilatation (31). Reverse left ventricular remodeling may occur after AVR with the improvement of diastolic function and reduction of LV hypertrophy and dilatation with MR improvement (32). In these patients, it is recommended to treat AS before mitral defect, as a sudden increase in afterload associated with MR repair may lead to cardiac decompensation. According to current guidelines (33) in patients with severe MR undergoing surgical AVR, mitral valve intervention is recommended. Following AVR, it is possible to reassess the severity of MR after a period of optimal medical therapy, considering the option of future mitral valve repair or replacement in the case of worsening of MR or persistence of symptoms (34, 35). No increased risk or technical complexity of MitraClip in the presence of prior TAVI has been described so far, assuming there is no significant distortion of the mitral valve annulus (30). However, when the left atrium is severely enlarged, a procedure targeting the mitral valve annulus, such as restrictive annuloplasty, may be appropriate. Atrial fibrillation, ventricular dyssynchrony, or prosthesis–patient mismatch are other causes of lack of improvement of MR after AVR (36). Interestingly, the use of self-expanding valves seems to be associated with less improvement in MR than balloon-expandable valves probably for the higher necessity of postoperative left bundle branch block and pacemaker insertion associated with self-expanding valves and the minor interference with mitral leaflet excursion annulus (37). A large prospective study is needed in order to define clear recommendations.

## Aortic Stenosis and Tricuspid Regurgitation (AS-TR)

TR is common in patients with left-side valvular disease and more specifically in 40% of patients with severe degenerative AS (38). Elevated left ventricular diastolic dysfunction and increased left atrial pressures due to AS reflect backward pressure to the pulmonary veins with the remodeling of the alveolar–capillary membrane and development of pulmonary hypertension (39). Chronic pulmonary congestion and pulmonary hypertension increase right ventricular afterload, and TR is caused by right atrium dilatation and leaflet mal-coaptation (40, 41). Eventually, these changes may promote atrial fibrillation, a condition *per se* associated with TR. In very advanced disease, backward flow and right ventricular dysfunction may cause a low-flow condition. For many years, the tricuspid valve has been regarded as the “forgotten valve” with a limited impact on hemodynamics, and management was conservative (42).

However, in the past decades, TR was found to have a negative prognostic impact in patients undergoing AVR (43–46). It is still not clear if TR is directly related to mortality or is a marker of advanced underlying disease (47). Patients with associated TR and AS usually are sicker with more comorbidities, more symptoms, and worse outcomes in terms of heart failure, hospitalization, and mortality. It is still debated if these patients would benefit from a combined intervention such as AVR and tricuspid annuloplasty or transcatheter tricuspid valve procedure (48) (Figure 1).

### Diagnostic Implications

TR severity, when functional in origin, closely depends on changes in loading condition. Thus, pre-operatory assessment is recommended (49).

Usually, none of the parameters used to evaluate AS or TR are reciprocally influenced; however, when TR is chronic and severe, a low-flow condition may superimpose, making it challenging to assess AS severity also with a classical continuity equation. In this setting, also an invasive evaluation with thermodilution method may underestimate the calculated aortic valve area and overestimate AS severity (50). Of note, these patients are usually symptomatic, and prognosis is very poor (51). It should be accounted for in case of severe TR: the reduced afterload may conceal an RV dysfunction; for this reason, contractility indexes should be higher than normal to exclude right-ventricle damage (Figure 1).

### Management

Successful correction of AS is associated with a long-term improvement in TR in 15–30% of patients. In some cases, TR may even worsen, suggesting that the mechanism is not entirely understood (52).

On the other hand, conservative therapy has a poor prognosis (53, 54). According to guidelines, tricuspid valve intervention should be considered (33) especially in the presence of tricuspid annular dilatation or signs of right heart failure (52).

Some studies suggest that tricuspid valve repair performed in a selected population undergoing left-sided valve surgery reduces mortality (55).

In the TAVI era, it is more difficult to entail tailored treatment and identify patients who would benefit from a combined intervention. Indeed, simultaneous moderate–severe TR results in an independent predictor of mortality despite multivariable adjustment, only in patients without MR and in patients with ejection fraction >30% (44, 47). Furthermore, additional intervention for TR should be evaluated based on right ventricular function and progression: TR after TAVI has shown to improve in 15–30% of patients (44, 46). In a retrospective study, surgical AVR combined with tricuspid valve repair and TAVI was both associated with a superior reduction in the TR jet area after 6 months compared with conservative therapy. However, right ventricular function improved after TAVI but not after surgical AVR+tricuspid valve repair, without a significant mortality difference (56).

Nowadays, both transcathether tricuspid valve repair is available and includes ring annuloplasty (56), spacer, MitraClip (57), and the TriClip device, which recently showed to be safe and effective at reducing TR by at least one grade in the Triluminate trial, where isolated TR was treated (58).

## Aortic Stenosis and Mitral Stenosis (AS-MS)

The association of MS and AS may be rheumatic in origin or the combination of degenerative calcific disease-causing hypomobility of the mitral valve leaflets and the aortic cusps. Finally, a variety of these causes is possible, especially in the presence of bicuspid aorta or radiation injuries (59, 60). In patients undergoing TAVI, concomitant MS is reported in 18% of patients (60).

The combination of double-valve stenosis in series is poorly hemodynamically tolerated, and usually, patients become symptomatic at an early stage of the disease (61). Mitral stenosis may severely impair preload and left ventricular filling, already damaged in a hypertrophic left ventricle, leading to a reduction in cardiac output and a paradoxical low-flow condition (62). Thus, the presence of AS may be somewhat masked. Clinical findings do not help as generally these patients present with dyspnea, a very vague symptom. Also, severe left atrial enlargement and atrial fibrillation are very common in this population.

However, the recognition of this double-valve pathology has important clinical implications as a correction of severe MS without treating AS first could impose a sudden increase in filling pressure to a small and hypertrophic left ventricle resulting in pulmonary edema.

Degenerative MS usually has a slower course than rheumatic, milder in severity MS and generally affects the aging population (63). The presence of MS associated with AS impacts mortality following both surgical AVR and TAVI (60, 64). Also, the presence of mild MS without documented secondary pulmonary hypertension or manifest valvular atrial fibrillation has a negative prognostic impact on TAVI (64) (Figure 1).

### Diagnostic Implications

As already mentioned, the low-flow, low-gradient condition may conceal an underlying AS if solely Doppler measurements are considered. Aortic valve morphology and planimetry may be helpful in identifying the underlying stenosis. The pressure half-time method that depends on the pressure difference between two chambers is not reliable due to the altered compliance of the left ventricle overestimating the mitral valve area (65, 66).

The continuity equation for the calculation of the aortic valve area and mitral valve area is not reliable because of its dependency on flow conditions, resulting in overestimation of MS in the setting of severe AS (67). By note, after AVR and normalization of the stroke volume, improvement of the mitral valve area has been described in almost half of the patients (68, 69), confirming that even pseudo severe mitral stenosis exists. For this reason, in these patients, where transaortic gradients may be low across both the mitral and aortic valves, 2D and 3D planimetry has a crucial role, and transesophageal echocardiography is often necessary as long as calcification does not impair the image quality.

The proximal isovelocity surface area method remains useful to quantify the mitral valve area when feasible. Sometimes, echocardiographic evaluation may not be exhaustive and cardiac catheterization may be necessary. Again, when severe MS significantly impairs cardiac output creating a low-flow low-gradient condition, the aortic valve area calculated with the Gorlin formula may result overestimated (Figure 1).

### Management

According to the current guidelines, bi-valvular surgery is indicated in the presence of MVA ≤ 1.5 cm^2^. Compared to isolated AVR, double valvular surgery is associated with higher operative mortality and poorer long term. According to Asami et al., even though MS was mild in the majority of cases and did not result in secondary pulmonary hypertension or manifest valvular atrial fibrillation, it was associated with a significantly worse prognosis. Rheumatic etiology showed an early higher incidence of adverse events than degenerative MS, probably due to a higher proportion of advanced stages of MS in patients with rheumatic MS (70).

Balloon dilatation may not be helpful in mitral calcific degenerative disease and can be dangerous in the case of annular calcifications (70). In patients at high surgical risk and not suitable for balloon valvuloplasty (71), trans-catheter mitral valve replacement is now possible with proven efficacy and safety (72, 73), also in combination with TAVI or subsequently (74). Yoon et al. compared the outcomes of the off-label use of TAVI devices in mitral annular calcification (ViMAC) for mitral stenosis, valve-in-valve (ViV), and valve-in-ring (ViR) procedures. ViMAC procedures showed a lower rate of technical success and a higher rate of all-cause mortality at the 30-day and 1-year follow-up (75).

In light of these data, the decision should be case-dependent, with concerns on anatomical and clinical features.

## Aortic Stenosis and Aortic Regurgitation (AS-AR)

The combination of AS and AR is part of the mixed aortic valve disease. About 75% of patients with a primary diagnosis of AS have some degree of concomitant AR. Conversely, 17.9% of patients with predominant AR have AS (2). Combined AS and AR are frequently observed in cases of bicuspid anatomy and rheumatic heart disease. Current guidelines are based primarily on the natural history of isolated AS or AR. Therefore, it is difficult to select those patients who could benefit from early valvular surgery. Nowadays, the management of this condition is determined by the severity of the dominant lesion. However, this approach is certainly oversimplifying (71).

AS causes ventricular hypertrophy while the regurgitation causes volume overload and ventricular dilatation, resulting in eccentric hypertrophy depending on the severity of each lesion (76). In case of significant AR, diastolic pressure is elevated with left ventricle filling on a steeper portion of the pressure–volume curve, potentially causing earlier onset of symptoms than if concomitant AR are not present (77).

In order to keep up with the elevated stroke volume, the ventricle progressively dilatates, producing an increasing wall tension that further worsens dilatation and reduces coronary perfusion (78). All these factors may explain earlier symptoms (79). On the other hand, compared to pure AR, concomitant AS limits the degree of LV dilation in response to the volume overload and the progression of AS tends to be slower.

Of note, in these patients AS severity has a predominant role in clinical outcomes (76).

This may suggest that a small degree of AR may put the severity of the valve disease at a higher stage, meaning strict monitoring for patients with moderate mixed aortic valve disease similar to those with isolated severe AS (Figure 1).

### Diagnostic Implications

In these patients, the increased stroke volume produces a higher transvalvular gradient that may overestimate AS. However, peak aortic jet velocity and mean gradient may still help to estimate the severity of AS and have a prognostic impact (76, 80). Furthermore, a simplified Bernoulli formula should not be used due to high left ventricular outflow tract velocities.

A continuity equation can be used with caution considering the high stroke volume. Furthermore, the left ventricular outflow tract geometry might not permit an accurate measure (79, 81). Of note, the Doppler velocity index is not significantly affected by the presence of AR.

The severity of AR can be assessed with ERO calculation and vena contracta jet as long as image quality permits. The presence of AS is also a confounder in assessing AR severity. Pressure half-time is not reliable because of ventricular diastolic function impairment, and when calculating the regurgitation volume, it must be remembered that in these patients, LV volume is smaller than in those with pure AR; thus, for any calculated regurgitant volume, the regurgitant fraction is higher (82). Eventually, in some cases, planimetry might be helpful, especially when other associated valvulopathies are suspected (83) (Figure 1).

### Management

The severity of AS and AR is correlated with prognosis and predicts the time to surgery (77).

Ong and Pibarot propose an algorithm for diagnosing and managing these patients that considers echocardiographic parameters, dobutamine stress test, CT calcium score, and clinical severity (84).

In patients with preserved ejection fraction and more than moderate AS and AR, AVR is found to improve morbidity and mortality when compared to medical therapy alone. Furthermore, in these patients, aortic valve area and aortic valve peak gradient progress faster than LV dilatation (85). Ideally, surgery should be done before developing ventricular dilatation and dysfunction because transcathether treatment is available; surgical risk in these patients is higher than in those with isolated AS (78). In mixed aortic valve disease patients, TAVI is associated with higher rates of paravalvular AR that, on the other hand, is generally well hemodynamically tolerated as the left ventricle is “preconditioned” to a volume overload (86).

## Conclusions

The presentation of aortic valve stenosis in the context of multiple valve disease is a highly prevalent condition, and it will increase over time with the aging population.

The hemodynamic interactions between AS and other valve diseases depend on the specific combination of valve lesions and may result in diagnostic pitfalls at echocardiography; therefore, other imaging modalities may be helpful. The heart valve team must integrate various parameters into the diagnosis and management strategy, including suitability for single or multiple transcatheter valve procedures.

## Author Contributions

All authors listed have made a substantial, direct and intellectual contribution to the work, and approved it for publication.

## Conflict of Interest

The authors declare that the research was conducted in the absence of any commercial or financial relationships that could be construed as a potential conflict of interest.

## Publisher's Note

All claims expressed in this article are solely those of the authors and do not necessarily represent those of their affiliated organizations, or those of the publisher, the editors and the reviewers. Any product that may be evaluated in this article, or claim that may be made by its manufacturer, is not guaranteed or endorsed by the publisher.
